# Isobutyraldehyde production from *Escherichia coli* by removing aldehyde reductase activity

**DOI:** 10.1186/1475-2859-11-90

**Published:** 2012-06-25

**Authors:** Gabriel M Rodriguez, Shota Atsumi

**Affiliations:** 1 Department of Chemistry, University of California, One Shields Ave, Davis, CA, 95616, USA

## Abstract

**Background:**

Increasing global demand and reliance on petroleum-derived chemicals will necessitate alternative sources for chemical feedstocks. Currently, 99% of chemical feedstocks are derived from petroleum and natural gas. Renewable methods for producing important chemical feedstocks largely remain unaddressed. Synthetic biology enables the renewable production of various chemicals from microorganisms by constructing unique metabolic pathways. Here, we engineer *Escherichia coli* for the production of isobutyraldehyde, which can be readily converted to various hydrocarbons currently derived from petroleum such as isobutyric acid, acetal, oxime and imine using existing chemical catalysis. Isobutyraldehyde can be readily stripped from cultures during production, which reduces toxic effects of isobutyraldehyde.

**Results:**

We adopted the isobutanol pathway previously constructed in *E. coli*, neglecting the last step in the pathway where isobutyraldehyde is converted to isobutanol. However, this strain still overwhelmingly produced isobutanol (1.5 g/L/OD_600_ (isobutanol) vs 0.14 g/L/OD_600_ (isobutyraldehyde)). Next, we deleted *yqhD* which encodes a broad-substrate range aldehyde reductase known to be active toward isobutyraldehyde. This strain produced isobutanol and isobutyraldehyde at a near 1:1 ratio, indicating further native isobutyraldehyde reductase (IBR) activity in *E. coli*. To further eliminate isobutanol formation, we set out to identify and remove the remaining *IBR*s from the *E. coli* genome. We identified 7 annotated genes coding for IBRs that could be active toward isobutyraldehyde: *adhP*, *eutG*, *yiaY*, *yjgB*, *betA*, *fucO*, *eutE*. Individual deletions of the genes yielded only marginal improvements. Therefore, we sequentially deleted all seven of the genes and assessed production. The combined deletions greatly increased isobutyraldehyde production (1.5 g/L/OD_600_) and decreased isobutanol production (0.4 g/L/OD_600_). By assessing production by overexpression of each candidate *IBR*, we reveal that AdhP, EutG, YjgB, and FucO are active toward isobutyraldehyde. Finally, we assessed long-term isobutyraldehyde production of our best strain containing a total of 15 gene deletions using a gas stripping system with *in situ* product removal, resulting in a final titer of 35 g/L after 5 days.

**Conclusions:**

In this work, we optimized *E. coli* for the production of the important chemical feedstock isobutyraldehyde by the removal of IBRs. Long-term production yielded industrially relevant titers of isobutyraldehyde with *in situ* product removal. The mutational load imparted on *E. coli* in this work demonstrates the versatility of metabolic engineering for strain improvements.

## Background

The dependence on finite petroleum and natural gas resources as well as their potential environmental impact has generated interest in exploring renewable sources for replacements. This has more notably been applied to the areas of transportation fuels. However, less attention has been paid to the chemical feedstock industry. Currently, 99% of chemicals and their derivatives come from petroleum and natural gas [1]. In 2004, the petrochemical industry consumed 4 quadrillion BTUs (British thermal units) of petroleum and natural gas for feedstock use to produce thousands of chemicals [1]. These chemicals are essential to the synthesis of plastics, rubbers, and pharmaceutical compounds that play a major role in our standard of living.

Synthetic biology has made large progress constructing pathways for the production of various biofuels [2-5]. Recently, these efforts have expanded to address the need for replacement of our current petrochemical feedstocks with renewable sources [6-13]. Much like in the production of advanced biofuels, synthetic biology offers a potential platform for the production of non-natural chemical feedstocks from simple sugars. This work aims to demonstrate the feasibility of producing chemical feedstocks from microorganisms by engineering *Escherichia coli* to produce isobutyraldehyde.

Isobutyraldehyde is used as both a fragrance and flavor additive. It is also used to produce plasticizers, isobutyric acid, and isobutanol, which is a precursor to the rubber polyisobutylene. Isobutyraldehyde is currently synthesized from petroleum derived propylene, carbon monoxide, and hydrogen [14]. In terms of microbial production and purification, its high volatility (172 mm Hg at 25°C) may enable less costly purification and facilitate *in situ* product removal for long-term fermentation.

**Figure 1:**
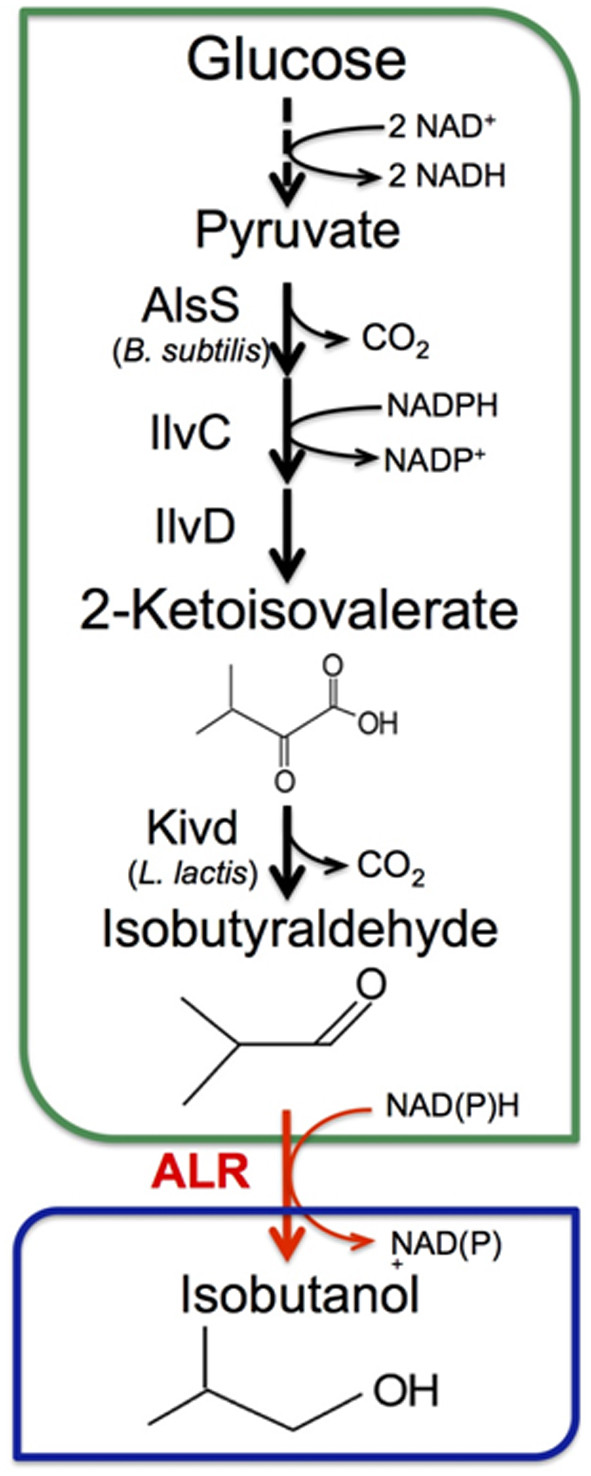
**Schematic representation of Isobutyraldehyde production in *E. coli*.** After glycolysis, two molecules of pyruvate are condensed into 2-acetolactate by acetolactate synthase (AlsS; *B. subtilis*), which is then converted into 2-ketoisovalerate (KIV) by acetohydroxy acid isomeroreductase and dihydroxy acid dehydratase (IlvC and ilvD; *E. coli*). Then, KIV is decarboxylated to form isobutyraldehyde by keto acid decarboxylase (Kivd; *L. lactis*). The target product, isobutyraldehyde, can be converted to the side product isobutanol by various aldehyde reductases (ALR) in *E. coli*.

Atsumi *et al.* have previously shown that 2-ketoisovalerate generated from L-valine biosynthesis can serve as precursors for the Ehrlich degradation pathway [15] to isobutanol (Figure 1) [5]. In this pathway, 2-ketoisovalerate is converted to isobutyraldehyde using a keto acid decarboxylase (KDC) and then reduced to isobutanol with an isobutyraldehyde reductase (IBR). In 2010, Atsumi *et al.* evaluated the native aldehyde reductase YqhD for its ability to convert isobutyraldehyde to isobutanol in *E. coli*[16]. It was discovered that a strain lacking overexpression of *IBR* was still able to produce the same amount of isobutanol as a strain overexpressing *ADH2* (*Saccharomyces cerevisiae*)*.* It was determined that YqhD in *E. coli* was responsible for most of IBR activity in isobutanol production. However, even with deletion of *yqhD* on the *E. coli* chromosome, the engineered strain was still able to produce isobutanol. It indicates that *E. coli* has one or several additional IBRs. Thus, here, we systematically removed possible *IBR*s from the *E. coli* chromosome to increase isobutyraldehyde production and reduce isobutanol formation.

## Results and discussion

As a starting point, *E. coli* strain JCL260 was used to assess initial isobutyraldehyde production. This strain was previously optimized for isobutanol production [5,17], by deleting *adhE, ldhA, fnr, frdAB, pta,* and *pflB*. The isobutyraldehyde pathway was constructed by using the previously described genes *alsS, ilvC, ilvD*, and *kivd*[5,18]*.*

To assess the initial production, pGR03 (*alsSilvC*, and *ilvD*) and pSA129 (*kivd*) were introduced into JCL260. This strain produced only 0.14 g/L/OD_600_ isobutyraldehyde, and as high as 1.45 g/L/OD_600_ isobutanol after 24 hours. This roughly 1:10 ratio is likely the result of one or several *IBR*s including *yqhD* on the *E. coli* genome [16]. As a result, we first deleted *yqhD*. This strain produced ~0.7 g/L/OD_600_ of isobutyraldehyde and ~0.4 g/L/OD_600_ isobutanol in our condition. This result indicates one or more additional *IBR*s exist on the *E. coli* genome.

IBR activity can be catalyzed by the alcohol dehydrogenase (ADH)/aldehyde reductase class of enzyme. Thus, in order to decrease isobutanol production, we searched the *E. coli* genome sequence using the EcoCyc comprehensive database [19] for other ADH-like enzymes. We identified seven candidate *IBR*s (*adhP, eutG, yiaY, yjgB, betA, fucO,* and *eutE*). Due to either the characterized ADH functions or the conserved ADH-like domain sequences of the above-mentioned genes, we hypothesized that these had the highest probability of having IBR activity. Additionally, no other ADH type enzymes were found. AdhP is a well-characterized NADH-dependent ethanol dehydrogenase [20]. EutG is an NADH-dependent alcohol dehydrogenase involved in ethanolamine utilization, and is suspected to be an ethanol dehydrogenase [21-23]. The enzymes EutG, YiaY, BetA, and EutE were previously utilized or removed to produce C4-C10 alcohols [24]. FucO has also been well characterized as a L-lactaldehyde and 1,2-propanediol oxidoreductase [25]. YjgB is a putative Zn-dependent aldehyde reductase [26]. With seven candidate genes identified, we set out to efficiently identify which (if any) of these enzymes were active toward isobutyraldehyde in order to improve isobutyraldehyde production and reduce isobutanol formation.

### Single gene deletions to improve isobutyraldehyde production

**Figure 2:**
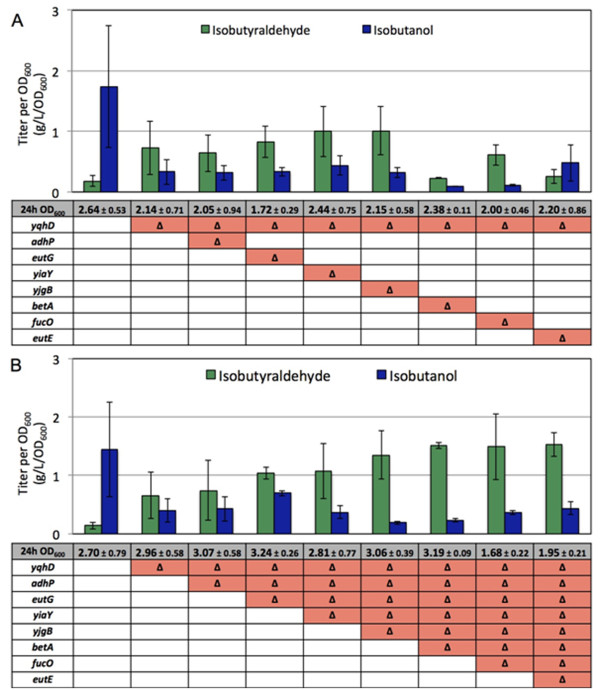
**Effects of individual and combined deletions of aldehyde reductases.** Cells were grown at 37°C for 24 h. ”Δ” indicates gene deletion. All strains contained pGR03 (*alsS, ilvC,* and *ilvD*) and pSA129 (kivd). Titers represented as concentration per OD_600_ to adjust for variations in growth. Error bars represent the standard deviation of triplicate experiments.

As a first step, each candidate *IBR* gene was individually deleted on the Δ*yqhD* strain (SA542 (Table 1)). Then, we measured production by introducing pGR03 (*alsS*, *ilvC*, and *ilvD*) and pSA129 (*kivd*) into each strain (Figure 2A). Production was carried out in sealed screw-cap culture tubes to prevent evaporation of isobutyraldehyde. None of the individual genes stood out as largely responsible for the observed isobutanol production (Figure 2A). However, two strains (Δ*yiaY* and Δ*yjgB*) showed slight improvements in isobutyraldehyde (up to 1 g/L/OD_600_) over the Δ*yqhD* strain (0.7 g/L/OD_600_), while two (Δ*betA* and Δ*eutE*) showed decreases to below 0.4 g/L/OD_600_. We considered that multiple enzymes could be involved in IBR activity, resulting in minor or no observable changes in production. In order to test this hypothesis, we set out to combine the deletions and assess production with each additional deletion. If several enzymes were responsible, then total elimination of these genes would result in higher isobutyraldehyde production, and little or no isobutanol formation.

**Table 1:** Strain and plasmids used in this work

**Name**	**Genotype**	**Reference**
BW25113	*rrnB*_T14_ Δl*acZ*WJ16 *hsdR514* Δ*araBAD*_AH33_ Δ*rhaBAD*_LD78_	[27]
JCL16	BW25113/F’ [traD36, proAB^+^, lacI^q^ ZΔM15]	[17]
JCL260	JCL16: Δ*adhE*; Δ*frdBC*; Δ*pta*; *Δfnr-ldhA*; Δ*pflB*	[5]
SA542	JCL260: Δ*yqhD*	[16]
AL287	SA542: Δ*adhP*	This work
AL288	SA542: Δ*eutG*	This work
AL289	SA542: Δ*yiaY*	This work
AL290	SA542: Δ*yjgB*	This work
AL555	SA542: Δ*betA*	This work
AL615	SA542: Δ*fucO*	This work
AL616	SA542: Δ*eutE*	This work
AL312	SA542: Δ*adhP*Δ*eutG*	This work
AL322	SA542: Δ*adhP*Δ*eutG*Δ*yiaY*	This work
AL329	SA542: Δ*adhP*Δ*eutG*Δ*yiaY*Δ*yjgB*	This work
AL556	SA542: Δ*adhP*Δ*eutG*Δ*yiaY*Δ*yjgB*Δ*betA*	This work
AL626	SA542:Δ*adhP*Δ*eutG*Δ*yiaY*Δ*yjgB*Δ*betA*Δ*fucO*	This work
AL707	SA542:Δ*adhP*Δ*eutG*Δ*yiaY*Δ*yjgB*Δ*betA*Δ*fucO*Δ*eutE*	This work
**Plasmids**	**Features**	**Reference**
pSA69	p15A ori; Kan^R^; *P*_L_lacO_1_: *alsS-ilvCD*	[5]
pSA129	ColE1 ori; Amp^R^; *P*_L_lacO_1_: *kivd*	[16]
pSA138	ColE1 ori; *P*_L_lacO_1_: *kivd-yqhD*	[5]
pGR03	p15A ori; Cm^R^; *P*_L_lacO_1_: *alsS-ilvCD*	This work
pAL217	ColE1 ori; Amp^R^; *P*_L_lacO_1_: *kivd-adhP*	This work
pAL218	ColE1 ori; Amp^R^; *P*_L_lacO_1_: *kivd-eutG*	This work
pAL219	ColE1 ori; Amp^R^; *P*_L_lacO_1_: *kivd-yiaY*	This work
pAL220	ColE1 ori; Amp^R^; *P*_L_lacO_1_: *kivd-yjgB*	This work
pAL221	ColE1 ori; Amp^R^; *P*_L_lacO_1_: *kivd-betA*	This work
pAL222	ColE1 ori; Amp^R^; *P*_L_lacO_1_: *kivd-fucO*	This work
pAL223	ColE1 ori; Amp^R^; *P*_L_lacO_1_: *kivd-eutE*	This work
pZE12-luc	ColE1 ori; Amp^R^; *P*_L_lacO_1_: *luc*(VF)	[28]
pAL162	ColE1 ori; Amp^R^; *P*_L_lacO_1_: *adhP*	This work
pAL158	ColE1 ori; Amp^R^; *P*_L_lacO_1_: *eutG*	This work
pAL157	ColE1 ori; Amp^R^; *P*_L_lacO_1_: *yiaY*	This work
pAL156	ColE1 ori; Amp^R^; *P*_L_lacO_1_: *yjgB*	This work
pAL213	ColE1 ori; Amp^R^; *P*_L_lacO_1_: *betA*	This work
pAL214	ColE1 ori; Amp^R^; *P*_L_lacO_1_: *fucO*	This work
pAL215	ColE1 ori; Amp^R^; *P*_L_lacO_1_: *eutE*	This work

### Combining gene deletions to improve isobutyraldehyde production

Next we sequentially combined all candidate *IBR* deletions. With each additional deletion we measured production of isobutyraldehyde and isobutanol (Figure 2B). The isobutyraldehyde production from the strain including Δ*adhP* Δ*eutG* and Δ*yiaY* was similar with that from the parent strain (Δ*yqhD*). However, with Δ*yjgB* added to the strain including Δ*yqhD* Δ*adhP* Δ*eutG* and Δ*yiaY* we observe a significant drop in isobutanol production to 0.19 g/L/OD_600_ and a significant increase in isobutyraldehyde production of 1.35 g/L/OD_600_. Subsequent deletions of Δ*betA*, Δ*fucO*, and Δ*eutE* on the strain including Δ*yqhD* Δ*adhP* Δ*eutG* Δ*yiaY* and Δ*yjgB* showed no additional reduction in isobutanol formation. Most strains showed similar growth after 24 hours to OD_600_ ~3, except for the last two deletions (Δ*fucO* and Δ*eutE*) which showed a marked decrease in 24 hour growth (OD_600_ ~ 1.7-2) (Figure 2B). The marked change in aldehyde ratio from 1:10 (JCL260) to as much as 7:1 (AL329(Δ*yqhD* Δ*adhP* Δ*eutG* Δ*yiaY* Δ*yjgB*)) represents a roughly ~10 fold increase in isobutyraldehyde production and ~7.5 fold decrease in isobutanol production (Figure 2B).

These results indicate that the remaining isobutanol production after deletion of *yqhD* was the result of multiple enzymes that have IBR activity. Additionally, despite deleting our entire list of *IBR* candidates in *E. coli*, there remains a small amount of isobutanol production. This suggests yet other enzymes are present that have IBR activity. Since no additional gene candidates were obvious in the *E. coli* genome and additional deletions became more difficult due to too many FLP recognition target sites [29], we did not search for further IBR candidates.

### Overexpression of candidate *IBR*s to confirm IBR activities

**Figure 3:**
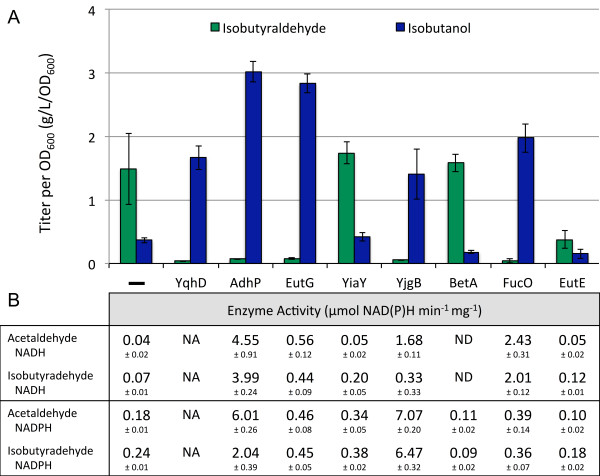
**Overexpression of each candidate IBR in AL626. (A)** Each candidate gene was cloned onto individual plasmids downstream of kivd (pAL213-pAL223) and introduced into AL626 (JCL260Δ*yqhD* Δ*adhP* Δ*eutG* Δ*yiaY* Δ*yjgB* Δ*betA* Δ*fucO*) along with pGR03 (*alsS, ilvC, and ilvD*). Cells were grown at 37°C for 24 h. Titers represented as concentration per OD_600_ to adjust for variations in growth. **(B)** Each gene was cloned onto individual plasmids and introduced into AL626. Cell extracts were assayed with acetaldehyde and isobutyraldehyde as substrates as well as with both cofactors (NADH & NADPH). Enzyme activity is defined as μmol NAD(P)H consumed per minute per mg of protein. NAD(P)H consumption measured at 340 nm. Error values represent the standard deviation of triplicate experiments. NA: not assayed. ND: not detectable.

In order to determine conclusively which of the candidate IBRs were active toward isobutyraldehyde, we took the low isobutanol producing strain AL626 (Δ*yqhD* Δ*adhP* Δ*eutG* Δ*yiaY* Δ*yjgB* Δ*betA* Δ*fucO*) and overexpressed each candidate *IBR* in addition to the isobutyraldehyde pathway. In this way, if the candidate IBRs are active, overexpression of these would reverse the ratio of isobutyraldehyde to isobutanol to levels similar to that of the parent strain (JCL260). The candidate *IBR*s were individually cloned onto pSA138, downstream of *kivd*, by replacing *yqhD* using SLIC (see Methods). The new plasmids along with pGR03 were then introduced into AL626. Production from the strains overexpressing *adhPeutGyjgB*, or *fucO* led to a reversal of production, while the other enzymes did not, indicating that these have IBR activity (Figure 3a). To verify proper expression of each enzyme from plasmid, cell extracts were ran on SDS-PAGE. All protein expressions were confirmed. The strain overexpressing *adhP* or *eutG* produced ~3 g/L/OD_600_, more than the strain overexpressing *yqhD* (1.7 g/L/OD_600_). This is likely because AdhP and EutG are NADH dependent [20,21,23], whereas YqhD is NADPH dependent [30]. The strain overexpressing *fucO* also produced slightly more than the strain overexpressing *yqhD* (2 g/L/OD_600_ vs 1.7 g/L/OD_600_), and is also NADH-dependent [25]. Since glycolysis produces two NADHs per one glucose, availability of NADH is generally higher in *E. coli* than NADPH. The strain overexpressing *yjgB* produced similar amounts of isobutanol as the strain overexpressing *yqhD*, verifying its putative annotation of aldehyde reductase/alcohol dehydrogenase [26] to be correct. The reason that deletion of *yqhD* has a greater affect than the other genes is likely due to higher expression levels from the genome. A comparison of mRNA levels from a transcriptome study show that mRNA levels of *yqhD* are between 2 and 10 fold higher than the other candidate genes, except for *betA*[31]. It is also known that the expression of *yqhD* is upregulated in the presence of aldehyde [32].

To further explore the activity of each IBR, enzyme assays were performed with each IBR candidate. We took the low isobutanol producing strain AL626 (Δ*yqhD* Δ*adhP* Δ*eutG* Δ*yiaY* Δ*yjgB* Δ*betA* Δ*fucO*) and overexpressed each candidate *IBR*. The candidate *IBR*s were individually cloned onto pZE12-*luc*, by replacing *luc* using SLIC (see Methods). The new plasmids and pZE12-*luc* (negative control) were then introduced into AL626. We assessed the activity of each enzyme with acetaldehyde (AA) and isobutyraldehyde (IBA) as substrates as well as with both cofactors, NADH and NADPH (Figure 3b). We confirmed aldehyde reductase activity of AdhP, EutG, YjgB, and FucO with both AA and IBA as substrates. The active enzymes showed similar preference for AA and IBA when assayed with their preferred cofactor. AdhP was able to utilize both NADH and NADPH, but IBR activity was 2-fold higher with NADH. FucO showed about 50% the activity of AdhP, consistent with production levels from Figure 3a. FucO showed clear preference for NADH, having ~6-fold higher activity with NADH than with NADPH for both aldehydes. The activity of EutG which is a Fe-containing ADH, as its sequence suggests [23], was not as high as compared to AdhP with AA (0.56 μmol NADH min^-1^ mg^-1^) and IBA (0.44 μmol NADH min^-1^ mg^-1^), but these activities were still 6 to 10-fold greater compared to the negative control. YjgB preferred NADPH and showed the highest activity of all the enzymes with both AA (7.07 μmol NADPH min^-1^ mg^-1^) and IBA (6.47 μmol NADPH min^-1^ mg^-1^). YiaY, BetA, and EutE showed very weak or no aldehyde reductase activity with both AA and IBA. The absence of activity from YiaY and BetA with AA and IBA is notable since they were previously used to produce > C5 alcohols [24].

### Long-term production with *in situ* product removal

**Figure 4:**
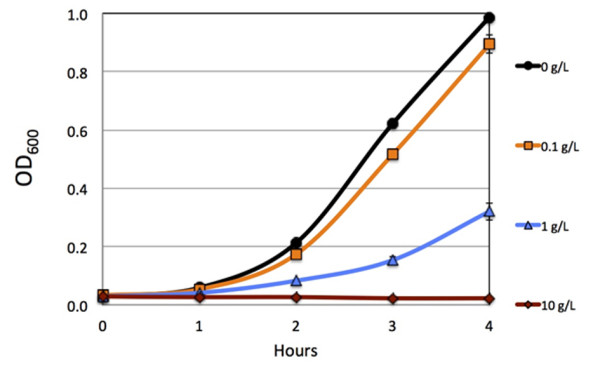
**Comparison of growth with isobutyraldehyde stress.** Time courses for the growth of E. coli strain AL626 (JCL260 Δ*yqhD* Δ*adhP* Δ*eutG* Δ*yiaY* Δ*yjgB* Δ*betA* Δ*fucO*) in the presence of 0 (black), 0.1 (orange), 1 (blue), and 10 g/L (red) isobutyraldehyde in 15 ml screw-cap tubes. Optical density (OD) measurements taken every hour for 4 hours at 600 nm. Error bars represent the standard deviation of triplicate experiments.

**Figure 5:**
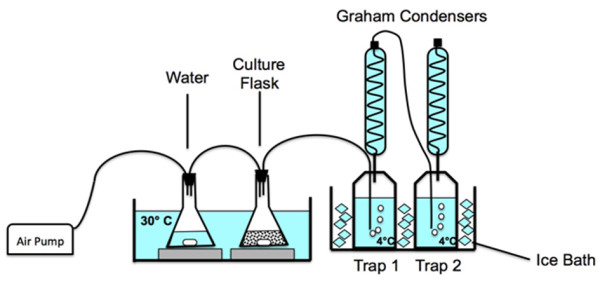
**Schematic of gas stripping system.** Sterile air is pumped (3 cc/min) into a flask containing 100 mL sterile water to saturate the air and thereby prevent evaporation of water in the culture flask. Vaporized product (isobutyraldehyde) is then carried into a series of traps and condensers held at 1 -4 °C where it is captured for quantification. Removal of product during production greatly reduces accumulation which is often toxic to the cells.

With a couple of high isobutyraldehyde producing strains, we explored the feasibility of long-term production of isobutyraldehyde. From toxicity experiments (Figure 4), we observe no growth at 10 g/L and significant growth inhibition with 1 g/L. Thus, we expect that toxicity of isobutyraldehyde accumulation could significantly hinder long-term production. In order to prevent inhibitory levels of aldehyde from accumulating, we applied a gas-stripping system to remove product *in situ* (Figure 5) [33]. Removal of product from a culture solution may also create a driving force for further product formation [34]. Furthermore, the redox balance of this pathway also necessitates sufficient supply of oxygen to the culture. Elimination of isobutanol formation negatively impacts the redox balance of the pathway under anaerobic conditions. Isobutanol formation results in a balanced redox state (Figure 1). As a result, isobutanol can be produced under anaerobic conditions. The isobutyraldehyde pathway, however, yields an excess of 1 NADH per glucose. This excess necessitates aerobic conditions in order to recycle the NAD^+^ pool through oxidative phosphorylation. Thus, using a gas-stripping system provides the cells with ample oxygen while simultaneously removing isobutyraldehyde from the culture.

In this set up, air flows through the culture flask, where vaporized isobutyraldehyde is captured in a series of cold (~4°C) condensers and ‘trap’ bottles. The high volatility of isobutyraldehyde (172 mmHg at 25°C) facilitates this process [14]. Since isobutyraldehyde is known to catalytically react with oxygen in the air between 30–50°C and convert to isobutyric acid [35], we conducted production at 30°C to minimize this. Furthermore, production of isobutanol at 30°C has been performed with greater success than at 37°C [34].

**Figure 6:**
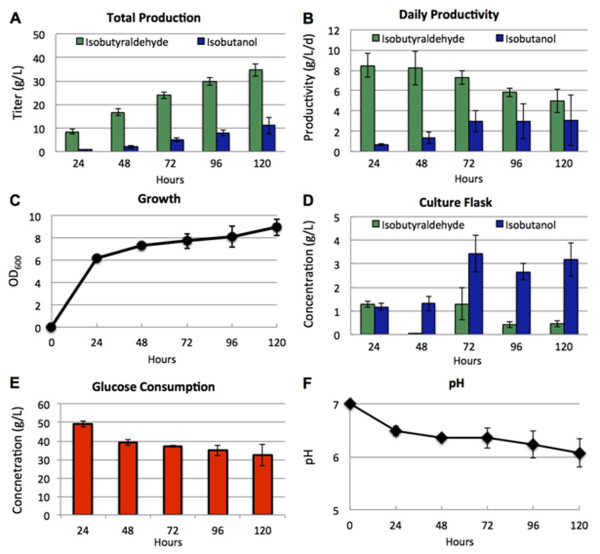
**Long-term production of isobutyraldehyde with a gas stripping system.** (**A**) Total accumulated production of isobutyraldehyde (green) and isobutanol (blue). (**B**) Daily productivity of isobutyraldehyde (green) and isobutanol (blue). (**C**) Time profiles of cell growth. (**D**) Concentrations of isobutyraldehyde (green) and isobutanol (blue) in the production culture to determine effectiveness of product removal by the system. (**E**) Daily glucose consumption of the culture. (**F**) Time profile of pH of the production culture. pGR03 (*alsS, ilvC,* and *ilvD*) and pSA129 (*kivd*) were introduced to AL626. Cells were inoculated 1% (vol/vol) from the overnight culture and grown in 100 mL production media (M9 media containing 5 g/L yeast extract and 50 g/L glucose). Every 24 hours, 10% of culture was removed for analysis, and replaced with production media containing 370 g/L glucose (dilutes to 37 g/L/d). Error bars represent the standard deviation of triplicate experiments.

Based on the results from Figure 3, we used AL626 for long-term production experiments (Figure 6). Production was carried out in a 250 mL baffled shake-flask under constant stirring. Cultures were initially grown at 37°C for faster growth and induced at OD_600_ ~0.4. Upon induction, the temperature of the culture bath was quickly brought down to 30°C. We optimized the airflow rate (3 cc/min) of the system such that the majority of isobutyraldehyde was collected in the first trap bottle and with minimal amounts remaining in the culture flask. Every 24 hours, 10% of the culture was removed for growth, pH, and product analysis and replaced with fresh media containing ~370 g/L glucose. Trap bottles were also analyzed and replaced every 24 hours with new trap bottles.

In the first 24 and 48 hours of production, the strain averaged 8 g/L/day of isobutyraldehyde (Figure 6B) with low isobutanol formation (0.7-1.3 g/L/day). As production continued from 72 to 120 hours, we observed a consistent decline of isobutyraldehyde productivity from 8 g/L/day to an eventual 5 g/L/day. This notably corresponded with a similar decline in glucose consumption from ~37 g/L/day at 48 hours, down to 32 g/L/day at 120 hours (Figure 6F). Surprisingly, we observed a marked increase in daily isobutanol formation beginning at 72 hours. After 120 hours, production of isobutanol began to overtake that of isobutyraldehyde in some trials, suggesting an up regulation of one or more unknown IBRs. For this reason, we stopped production after 120 hours. The culture grew to OD_600_ 6.2 after the first day and increased slowly thereafter up to OD_600_ 9 after 120 hours (Figure 6C). This suggests the culture received sufficient oxygen to achieve redox balance throughout the production. The pH of the culture (Figure 6F) remained fairly stable throughout production, decreasing only by 1 unit to a pH of about 6 after 120 hours. In the future, maintaining pH at 7 by addition of base or by using a buffer may improve yield and culture stability with this system.

Overall, isobutyraldehyde levels in the culture flask remained low, typically between 0.5 g/L and 1.5 g/L (Figure 6D). The stable growth and relatively low product accumulation (Figure 6D), indicates the gas-stripping system was successful in reducing the potential toxicity of isobutyraldehyde. Development of an isobutyraldehyde tolerant strain may improve cell growth and culture stability during production, possibly circumventing the need for gas-stripping. Strains tolerant of isobutanol have previously been developed for similar reasons [36]. However, an increase in isobutanol tolerance did not correlate with improved production [34]. Our strain’s productivity of 8 g/L/day is roughly 8-fold greater than toxic levels of isobutyraldehyde (~1 g/L). Achieving a strain that can tolerate these titers may be difficult to develop. Additionally, utilizing gas-stripping has the added advantage of purifying isobutyraldehyde during production, which may be a cost saving method under industrial scale production.

Isobutyraldehyde production from this strain totaled 35 g/L after 120 hours (Figure 6A). Including formation of isobutanol (~10 g/L), total production reached ~45 g/L. The theoretical molar yield of isobutyraldehyde from glucose is 1:1. This results in a gram per gram yield of 0.40 g isobutyraldehyde/g glucose. In the first 48 hours of production, we achieved a yield of 47% of the theoretical maximum. The yield remained relatively constant throughout the experiment with a final yield of 45% of the theoretical maximum after 120 hours.

The reason for the large increase in isobutanol formation is not entirely obvious. Growth, pH, and overall yield remained stable throughout the experiment, but glucose consumption and isobutyraldehyde production slowly decreased. Despite the discovery and removal of five *IBR*s from the genome, still further significant IBR activity exists in our *E. coli* strain. One possibility is that expression of the unknown enzyme(s) is low early on in production and later upregulated. Cells that are able to increase expression of such enzyme(s) will likely experience lower toxicity and may begin to outgrow other cells within the culture. Thus, further exploration and elimination of this IBR activity will be needed to achieve more stable isobutyraldehyde production after 72 hours.

## Conclusions

In this work, we demonstrated the renewable production of isobutyraldehyde, an important chemical feedstock, from *E. coli*. We identified AdhP, EutG, YjgB, and FucO as isobutyraldehyde reductases, while YiaY, BetA, and EutE were not active toward isobutyraldehyde. With *in situ* product removal, we achieved a total isobutyraldehyde production of 35 g/L after 5 days. This industrially relevant titer represents a yield of 45% of the theoretical maximum. However, after the second day, isobutanol formation began to increase markedly, suggesting upregulation of yet additional *IBR*(s) in *E. coli.*

The lingering presence of isobutanol indicates that additional IBRs still exist in *E. coli* which we have yet to identify. In this work, we evaluated genes more specifically annotated or characterized as alcohol dehydrogenases. It is possible that other non-specific oxidoreductases are contributing to IBR activity in the cell. *E. coli* contains more than a dozen other putative, less specifically annotated, NAD(P)H dependent oxidoreductases. Going forward, applying transcriptome analysis, bioinformatics, and other methods may uncover the remaining IBR(s). Further exploration and elimination of these reductases will be required to improve the long-term production stability of this strain.

## Methods

### Reagents

Rapid Ligation Kit was purchased from Roche. All other enzymes were purchased from New England Biolabs (Ipswich, MA). All synthetic oligonucleotides were ordered from Integrated DNA Technologies (Coralville, IA). DNA sequencing services were done by Davis Sequencing (Davis, CA). Zymo DNA Clean and Concentrator kit and Gel Recovery kit (Zymo Research, Irvine, CA) were used to purify all PCR products. Isobutanol and Isobutyraldehyde were purchased from Sigma Aldrich (St. Louis, MO). 1-pentanol was purchased from Acros Organics (Belgium).

### Media

Overnight cultures were grown in 5 mL Luria Broth (LB) containing appropriate antibiotics. Antibiotic concentrations were as follows: Kanamycin (50 μg/mL), Chloramphenicol (40 μg/mL), Ampicillin 100 (μg/mL), Tetracycline (20 μg/mL). Production was carried out with M9 medium containing 5 g/L yeast extract, ~50 g/L glucose, and 1000-fold dilution of A5 trace metal mix (2.86 g H_3_BO_3_, 1.81 g MnCl_2_⋅4H_2_O, 0.222 g ZnSO_4_ ⋅7H_2_O, 0.39 g Na_2_MoO_4_⋅2H_2_O, 0.079 g CuSO_4_⋅5H_2_O, 49.4 mg Co(NO_3_)_2_⋅6H_2_O per liter water).

### Gene deletions

All gene deletions were carried out by P1 transduction [37] with the aid of strains from the Keio collection [29], except for *eutE*. To delete *eutE*, the method developed by Datsenko and Wanner [27] was used due to its proximity to *eutG*. The deleted fragments were verified by PCR and sequencing. All strains used in this study are listed in Table 1.

### Plasmid construction, cloning, and transformations

To construct pGR03, first pSA69 [5] was digested with AatII and SacI, and treated with Antarctic phosphatase. Then the chloramphenicol resistance gene was taken from pZA31-luc [28] by digestion with AatII and SacI. The two fragments were gel purified, ligated, and introduced into *E. coli* XL-1 blue strain.

All other plasmids (pAL156-158, pAL162, pAL213-215, pAL217-223) were constructed using sequence and ligation-independent cloning (SLIC) [38]. In general, primers for inserts (GR147-154, GR188-193, GR219-GR232) were designed with ~25 bp priming to the target gene and a 20–25 bp ‘linker’ to target vector. All candidate *IBR*s were amplified by PCR from *E. coli* JCL16 genome DNA. Plasmids were verified by colony PCR, by digestion with restriction enzymes, and by sequencing. All plasmids and oligonucleotides are listed in Table 1 and Table 2, respectively.

**Table 2:** Primer sequences

**Primer**	**Sequence 5’ → 3’**
GR180	TCTAGAGGCATCAAATAAAACGAAAGGCTC
GR181	GGTATATCTCCTGCATGCTTATGATTTATTTTG
GR219	GCATGCAGGAGAAAGGTCACatgaaggctgcagttgttacgaagg
GR220	TTTTATTTGATGCCTCTAGAttagtgacggaaatcaatcaccatg
GR221	GCATGCAGGAGAAAGGTCACatgcaaaatgaattgcagaccgcgc
GR222	TTTTATTTGATGCCTCTAGAttattgcgccgctgcgtacaggccg
GR223	GCATGCAGGAGAAAGGTCACatggcagcttcaacgttctttattc
GR224	TTTTATTTGATGCCTCTAGAttacatcgctgcgcgataaatcgcc
GR225	GCATGCAGGAGAAAGGTCACatgtcgatgataaaaagctatgccg
GR226	TTTTATTTGATGCCTCTAGAtcaaaaatcggctttcaacaccacg
GR227	GCATGCAGGAGAAAGGTCACAtgcaatttgactacatcattattg
GR228	TTTTATTTGATGCCTCTAGAtcattttttcgctctcaccggcatc
GR229	GCATGCAGGAGAAAGGTCACatgatggctaacagaatgattctga
GR230	TTTTATTTGATGCCTCTAGAttaccaggcggtatggtaaagctct
GR231	GCATGCAGGAGAAAGGTCACatgaatcaacaggatattgaacagg
GR232	TTTTATTTGATGCCTCTAGAttaaacaatgcgaaacgcatcgact
GR145	GGTACCTTTCTCCTCTTTAATGAA
GR146	TAATGACTCTAGAGGCATCAAATAA
GR147	ATTCATTAAAGAGGAGAAAGGTACCatgaaggctgcagttgttacgaagg
GR148	TTATTTGATGCCTCTAGAGTCATTAttagtgacggaaatcaatcaccatg
GR149	ATTCATTAAAGAGGAGAAAGGTACCatgcaaaatgaattgcagaccgcgc
GR150	TTATTTGATGCCTCTAGAGTCATTAttattgcgccgctgcgtacaggccg
GR151	ATTCATTAAAGAGGAGAAAGGTACCatggcagcttcaacgttctttattc
GR152	TTATTTGATGCCTCTAGAGTCATTAttacatcgctgcgcgataaatcgcc
GR153	ATTCATTAAAGAGGAGAAAGGTACCatgtcgatgataaaaagctatgccg
GR154	TTATTTGATGCCTCTAGAGTCATTAtcaaaaatcggctttcaacaccacg
GR188	ATTCATTAAAGAGGAGAAAGGTACCAtgcaatttgactacatcattattggtg
GR189	TTATTTGATGCCTCTAGAGTCATTAtcattttttcgctctcaccggcatc
GR190	ATTCATTAAAGAGGAGAAAGGTACCatgatggctaacagaatgattctgaac
GR191	TTATTTGATGCCTCTAGAGTCATTAttaccaggcggtatggtaaagctc
GR192	ATTCATTAAAGAGGAGAAAGGTACCatgaatcaacaggatattgaacaggtg
GR193	TTATTTGATGCCTCTAGAGTCATTAttaaacaatgcgaaacgcatcgact

Vector was amplified from pSA138 with GR180 and GR181, and digested with DpnI to reduce background associated from plasmid template. Primers (GR219 and GR221 for *adhP*, GR221 and GR222 for *eutG*, GR223 and GR224 for *yiaY*, GR225 and GR226 for *yjgB*, GR227 and GR228 for *betA*, GR229 and GR230 for *fucO*, and GR231 and GR232 for *eutE*) were used to amplify each gene from *E. coli* JCL16 genome DNA and cloned with SLIC onto the pSA138 vector fragment.

Vector was amplified from pZE12-luc with GR145 and GR146, and digested with DpnI to reduce background associated from plasmid template. Primers (GR147 and GR148 for *adhP*, GR149 and GR150 for *eutG*, GR151 and GR152 for *yiaY*, GR153 and GR154 for *yjgB*, GR188 and GR189 for *betA*, GR190 and GR191 for *fucO*, and GR192 and GR193 for *eutE*) were used to amplify each gene from *E. coli* JCL16 genome DNA and cloned with SLIC onto the pZE12-luc vector fragment.

### Screw-cap tube production

For isobutyraldehyde production test, 1% (vol/vol) of the overnight culture was inoculated in 5 mL production media in 15 mL screw-cap culture tubes and grown at 37°C in a rotary shaker (250 RPM) until OD_600_ ~0.4, then induced with 1 mM isopropyl-*β*-D-thio-galactoside (IPTG) and allowed to produce for 24 hours after induction. Screw-cap tubes were tightly sealed to prevent evaporation of isobutyraldehyde.

### Aldehyde reductase activity assay

The plasmids pZE12-luc, pAL162, pAL156-158, and pAL213-214 were introduced into AL626. The strains were grown to OD_600_ value of ~0.4 in 5 mL LB medium at 37°C, followed by adding 1 mM IPTG. Protein overexpression was performed at 37°C for 4 h. Then 1.8 mL of cells were centrifuged at 13,000 RPM for 10 minutes, resuspended in 300 μL BugBuster Protein Extraction Reagent (Novagen, San Diego, CA, USA), and incubated at room temperature for 20 min for cell lysis. The samples were centrifuged for 20 min, 16,000 g, at 4°C. Supernatants were taken for enzyme assays. ADH activities were measured by following the reduction of acetaldehyde or isobutyraldehyde with NADH or NADPH at 340 nm at 37°C using a Synergy H1 Hybrid Plate Reader from BioTek Instruments, Inc. (Winooski, VT). The assay mixture contained 25 mM acetaldehyde or isobutyraldehyde, 50 mM 3-(*N*-morpholino)propanesulfonic acid (MOPS) buffer (pH 7.0), 0.2 mM Tris-Cl (pH 7.00), 0.2 mM NAD(P)H, and 12.5 mM potassium phosphate buffer (pH 7.5). One unit of activity is defined as the oxidation of 1 μmol of NAD(P)H per minute per mg protein. Protein concentrations were measured using 5x Advanced Protein Assay Reagent (Cytoskeleton Inc., Denver, CO) by diluting 5μL of cell extract in 1 mL of 1x Advanced Protein Assay Reagent and measuring the OD_590_ of the mixture. Bovine Serum Albumin (BSA) from NEB was used to prepare a standard curve.

### Gas stripping apparatus

Rubber stoppers were used to cap the culture flask and water flask. Two holes were made in each rubber stopper where small, customized, metal pipes were inserted. All flasks, traps, and condensers were connected with clear, flexible, chemical resistant, plastic tubing (Tygon tubing). Air flow rate was set to 3 cc/min with a flow regulator. Trap bottles were filled with 600 mL of water. Culture volume and water flask volume were both 100 mL. Culture was initially grown at 37°C until OD_600_ ~0.4, then 1 mM IPTG was added and the temperature was quickly brought down to 30°C for the remainder of the experiment. When trap bottles were collected for samples, 200 mL water was washed down each condenser to rinse any product into the trap bottles. Final trap volume was determined with a 1000 mL graduated cylinder.

### GC analysis

Concentrations of all products, except glucose, were analyzed by Gas Chromatography equipped with a flame ionization detector (FID). The GC system is a Shimadzu GC-2010 with an AOC-20 S auto sampler and AOC-20i Auto Injector. The column used was a DB-Wax capillary column (30 m length, 0.32-mm diameter, 0.50-μm film thickness) from Agilent Technologies. GC oven temperature was initially held at 40°C for 3 minutes, then increased at a rate of 45°C min^-1^ until 235°C and held for 3 min. Injector temperature was held at 225°C and FID detector was held at 330°C. Injection volume was 0.5 μL, injected at a 15:1 split ratio. Helium was used as the carrier gas. 1-pentanol was used as internal standard.

### Glucose quantification and growth measurements

Glucose was measured using a Sucrose, Fructose, and Glucose kit from Megazyme International Unlimited. Glucose assays and Optical densities (OD) were measured at 340 nm and 600 nm, respectively, with a Genesis 10 S UV–vis Spectrophotometer (Thermo Scientific).

## Competing interests

A provisional patent regarding this technology has been filed.

## Authors’ contributions

S.A. and G.M.R designed experiments; G.M.R. performed the experiments; S.A. and G.M.R. analyzed the data; and S.A. and G.M.R. wrote the paper. All authors read and approved the final manuscript.
